# 

**Published:** 1909-05-15

**Authors:** 


### ANNOUNCEMENTS.

INTERNATIONAL DENTAL EXHIBITION. This is to be a convention of English dealers and manufacturers of dental supplies, and its exhibition will be held in Royal Horticultural Hall, Westminister, London, England, September 6th to 10th, 1909. It is intended to concentrate in this exhibit every article made for the use of the dentist, and all dentists are cordially invited to attend.

RETIREMENT FROM PRACTICE. Dr. E. C. Moore, of Detroit, announces his retirement from practice. Dr. Moore has had a long and enviable career as a practitioner of dentistry in Detroit. He graduated from the Ohio College of Dental Surgery in 1870, and has practiced in Detroit ever since. He has taken a large part in the professional work of the State and enjoyed one of the best practices in the State. He is succeeded in practice by his son, Lindsey.

THE ALUMNI ASSOCIATION, College of Dentistry University of California, and the California State Dental Association will hold a joint session on July 6th, 7th and 8th, at the College of Dentistry Building, First and Parnassus Aves., San Francisco.

Arrangements are being made which promise to make the session mark an epoch in Dental work on the coast. Dr.

John Q. Byram has been secured and attendance at his clinic will be equal to a post-graduate course in Porcelain.

Negotiations are being continued for one other eastern clinician with promise of success.

Save these three days for a most profitable meeting—the knowledge gained will amply repay you.

---

THE RANSOM & RANDOLPH CO, of Toledo, has purchased the dental supply business of the Cogswell Dental Co., of Cleveland, and will maintain a branch house in that city.

---

IOWA BOARD OF DENTAL EXAMINERS. The next meeting of the Iowa State Board of Dental Examiners for examination will be held at Iowa City, beginning June 7th, 1909, at 9:00 A. M.

Practical examination will be held in both Operative and Prosthetic Dentistry.

Applications must be in the hands of the secretary by June 1st. For further information address

E. D. BROWER, *Secretary*,

Le Mars, Iowa.

---

NATIONAL DENTAL ASSOCIATION. The thirteenth annual meeting of the National Dental Association, held at Birmingham, Ala., March 30th to April 2nd, was a most successful one with a good attendance.

The papers and discussions were exceedingly interesting and held the close attention of large audiences throughout.

Official action was taken providing for a National Dental Journal, commencing October 1910.

The committee on revision of Constitution and By-Laws presented a number of amendments embodying a liberal plan of re-organization. Copies carrying the proposed changes are to be printed and mailed to the membership at an early date, which will give ample opportunity to thoroughly understand same befor final action is taken.

The following officers were elected: President, Burton Lee Thorpe, St. Louis, Mo.; Vice President for the West, W. T. Chambers, Denver ,Colo.; Vice President for the East, Charles W. Rodgers, Boston, Mass.; Vice President for the South, Thomas P. Hinman,Atlanta, Ga.; Corresponding Secretary, H. C. Brown, Columbus, Ohio; Recording Secretary, Charles S. Butler, Buffalo, N. Y.; Treasurer, A. R. Melendy, Knoxville, Tenn.; Executive Committee, (new members for three years) :C. M. Werk, Ottumwa, Iowa; V. H. Jackson, New York City; W. G. Mason, Tampa, Fla. Executive Council: H. J. Burkhart, Batavia, N. Y.; B. Holly Smith, Baltimore, Md.; A. H. Peck, Chicago, Ill.; W. E. Boardman, Boston, Mass.; C. L. Alexander, Charlotte, N. C.

Denver Colorado, and the third Tuesday of July, 1910, were chosen as the place and date of the next meeting.

H. C. Brown, *Cor. Sec'y.*

